# Exosomes: a review of biologic function, diagnostic and targeted therapy applications, and clinical trials

**DOI:** 10.1186/s12929-024-01055-0

**Published:** 2024-07-11

**Authors:** Yi-Fan Chen, Frank Luh, Yuan-Soon Ho, Yun Yen

**Affiliations:** 1 International Master Program in Translation Science, College of Medical Science and Technology, Taipei Medical University, New Taipei City, 23564 Taiwan; 2 The Ph.D. Program for Translational Medicine, College of Medical Science and Technology, Taipei Medical University, New Taipei City, 23564 Taiwan; 3 TMU Research Center of Cancer Translational Medicine, Taipei Medical University, Taipei, 11031 Taiwan; 4 International Ph.D. Program for Translational Science, College of Medical Science and Technology, Taipei Medical University, New Taipei City, 23564 Taiwan; 5 Master Program in Clinical Genomics and Proteomics, School of Pharmacy, Taipei Medical University, Taipei, 11031 Taiwan; 6 Sino-American Cancer Foundation, Covina, CA 91722 USA; 7 Institute of Biochemistry and Molecular Biology, College of Life Sciences, China Medical University, Taichung, 406040 Taiwan; 8 Ph.D. Program for Cancer Molecular Biology and Drug Discovery, College of Medical Science and Technology, Taipei Medical University, Taipei, 110301 Taiwan; 9 Graduate Institute of Cancer Biology and Drug Discovery, College of Medical Science and Technology, Taipei Medical University, Taipei, 110301 Taiwan; 10 Cancer Center, Taipei Municipal WanFang Hospital, Taipei, 11696 Taiwan; 11 Center for Cancer Translational Research, Tzu Chi University, Hualien City, 970374 Taiwan

**Keywords:** Exosome, siRNA, Cancer diagnosis, Cancer therapy

## Abstract

Exosomes are extracellular vesicles generated by all cells and they carry nucleic acids, proteins, lipids, and metabolites. They mediate the exchange of substances between cells,thereby affecting biological properties and activities of recipient cells. In this review, we briefly discuss the composition of exocomes and exosome isolation. We also review the clinical applications of exosomes in cancer biology as well as strategies in exosome-mediated targeted drug delivery systems. Finally, the application of exosomes in the context of cancer therapeutics both in practice and literature are discussed.

## Introduction

**Fig. 1:**
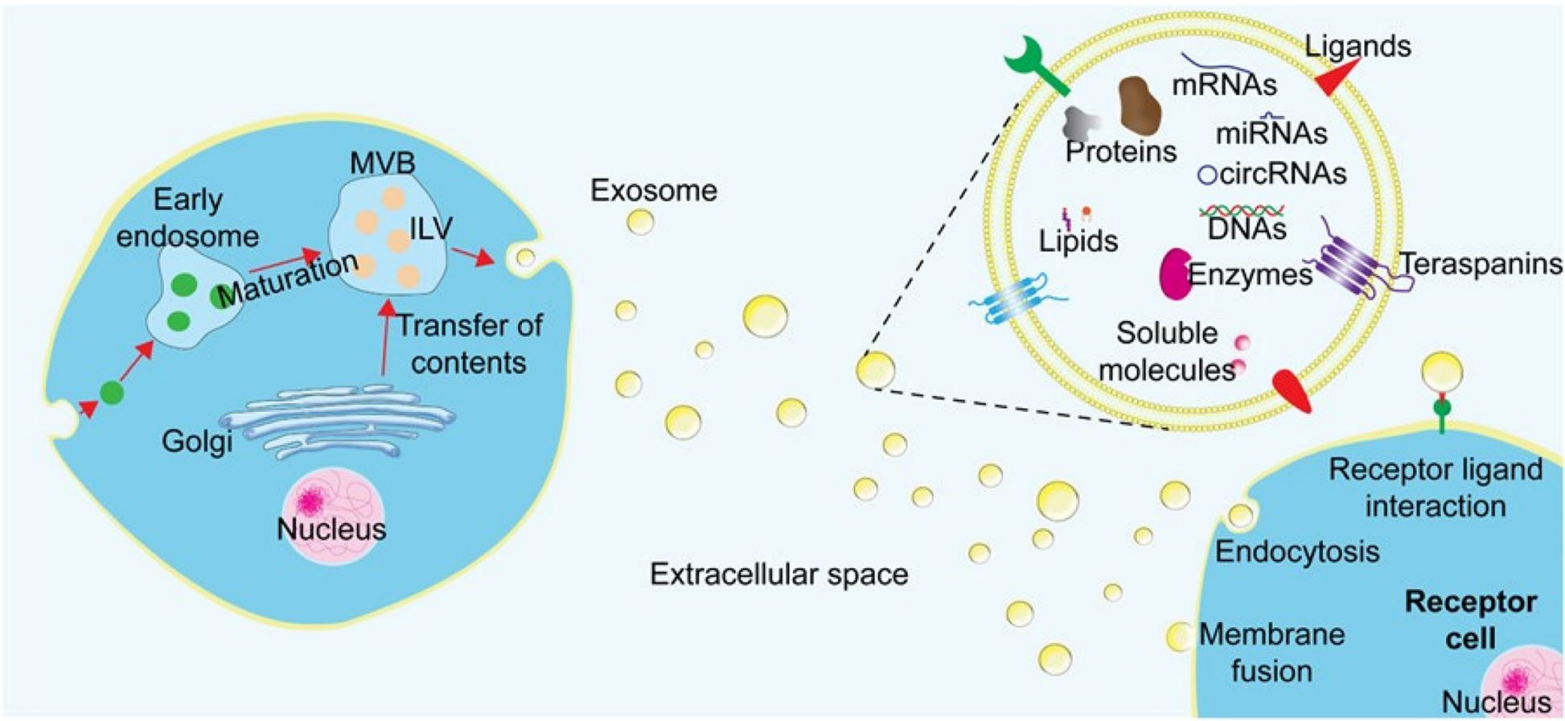
Biosynthesis and diversity of exosomes. In their early stage of formation in living cells, exosomes are produced through an endosomal pathway and released into the extracellular space. Many cellular contents, such as proteins, lipids, metabolites, small molecules, DNA, RNA, and cell membrane surface proteins, are enclosed in exosomes during this process and fuse with the recipient cells via endocytosis and plasma membrane invagination. Exosomes released from donor cells can be internalized through endocytosis or membrane fusion and can also trigger a biological response in the recipient cell through interaction with cell surface proteins or receptors

Exosomes are classified as small (30–150 nm), phospholipid bilayer extracellular vehicles released by both prokaryotic and eukaryotic cells for intercellular communication and signaling [1]. Historically, exosomes were considered extracellular vesicles that excrete unwanted cellular waste. However, further research has demonstrated that exosomes are important molecular mediators for cellular communication for transporting proteins, metabolites, and various nucleic acids throughout the body [2, 3] (Fig. 1). Exosomes are also secreted by a wide variety of cells, including immune cells [4], cancer cells [4], and stem cells [5]. Due to their multiple functions in transmitting information between cells, exosomes play a critical role in physiological regulation, disease progression, immune response, and disease development [6].

Exosomes are produced naturally within the human body and are not prone to immunogenicity, which would otherwise elicit a host response [7]. Clinically, they have been regarded as ideal candidates for applications related to biomarker development and early disease detection [8]. Furthermore, exosomes have garnered recent attention for their potential as drug delivery vehicle, which could improve factors like bioavailability of cargo load, side effect profiles, off-target effect, and pharmacokinetics for drug molecules [9, 10].

Due to their ability to transport cellular material systemically as well as reflect the physiological state of donor cells, exosomes can influence various processes related to inflammation, central nervous system communication, immune response, and different types of tissue repair [11]. Their phospholipid bilayer membrane contains lipid structures like ceramide and cholesterol, which assist in sorting, secretion, and signaling between host cell and extracellular environment [12]. The abundance of RNA species within exosomes plays a critical role in various biological processes, especially in different disease states and progression factors. For example, exosomes are involved in the transfer of pathogens such as virus particles [13] and nucleic acids such as circular RNAs (circRNAs) associated with tumor progression [14]. Some studies have shown that exosomes can serve as potential biomarkers for early diagnosis and monitoring of diseases like cancer [15–17]. Accordingly, exosomes may be considered an important therapeutic tool for treatment and early disease detection.

In this review, we provide an overview of the exosome field. We review the unique properties and diverse content of exosomes and current methods to isolate them. In addition, we discuss clinical applications of exosomes in the context of cancer biology. Exosome-mediated targeted drug delivery as well as a current case study also discussed. Finally, we provide an overview of exosomes currently under review in clinical trials and provide insight on future challenges.

## The complex architecture of exosomes and their isolation methods

Many constitutive elements have been identified in exosomes from different cell types— including approximately 4400 proteins, 194 lipids, 1639 mRNAs, and 764 miRNAs—illustrating their complexity and potential functional diversity [18]. Typically, exosomes are highly enriched proteins with multiple functions, such as transmembrane proteins (CD9, CD81, CD82), which take part in cell penetration, invasion, and fusion; heat shock proteins (HSP70, HSP90) for stress response in antigen binding and presentation; MVB formation proteins (TSG101 Alix) for exosome release; and proteins for membrane transport and fusion (annexin and Rab) [19, 20]. A list of protein components commonly found in exosomes is shown in Table 1 [21–23].

**Table 1:** Common protein components of exosomes

Protein Type and Function	Specific Example(s)
Heat Shock Proteins (HSP)	HSP90, HSP70, HSP27, HSP60
Cell Adhesion	Integrin, Lactadherin, Intercellular Adhesion Molecule
Antigen Presentation	Human Leukocyte Antigen Class I/II, Peptide Complexes
Signaling Proteins	HTPase, Ras, Src, GDP Dissociation Inhibitor, Syntenin1, 14–3-3 Proteins, Transforming protein RhoA
Iron Transport	Transferrin Receptor
Growth Factors and Cytokines	TNF α, Transforming growth factor β
Trafficking and membrane fusion	Ras-related protein, Annexin I, II, IV, V, VI, Dynamin, Syntaxin 3
Transcription and Protein Synthesis	Histone1, 2, 3, Ribosomal Proteins, Ubiquitin, Complement factor
Cytoskeletal components	Actin, Myosin, Tubulin, Vimentin, Radixin
**References**	[24–26]

In addition to select proteins, exosomes also contain multiple patterns of RNAs that can be incorporated into recipient cells. RNA sequencing analysis has demonstrated that microRNAs (miRs) are most abundant in human plasma-derived exosomal RNA species, making up over 42% of all raw reads and 76% of all mappable reads [27]. Other RNA species include ribosomal RNA, long non-coding RNA, piwi-interacting RNA, transfer RNA, small nuclear RNA, and small nucleolar RNA. Once miRs are packed into exosomes, they undergo unidirectional transfer between cells, resulting in an intercellular trafficking network, eliciting transient or persistent phenotypic changes of recipient cells [28]. Besides miRs, other proteins like long RNA species (e.g., long non-coding RNAs and circular RNAs) have also been identified in exosomes that impact various biological processes, including cancer development [29].

Exosomes are are not only classified according to protein and nucleic acids composition but also by their lipid components. Generally, exosomes are enriched in phosphatidyl-serine (PS), phosphatidic acid, cholesterol, sphingomyelin, arachidonic acid and related fatty acids, prostaglandins, and leukotrienes – all of which account for their stability and structural integrity (Table 2) [10, 30–34]. Moreover, exosomes also have some functional lipolytic enzymes, which produce bioactive lipids autonomously. These exosomal bioactive lipids may be internalized into recipient cells and concentrate lipid mediators within the endosomes. Recent studies have revealed that the accumulation of prostaglandins and fatty acids brought by exosomes can trigger the activation of cell-to-cell phospholipases and lipid mediators [33]. Because exosomes have been found to be commonly released and taken up by target cells to modulate cell lipid metabolism, studies have examined their role in lipid-related pathologies (atherosclerosis) as well as their potential role as a molecular signal for disease diagnosis and prognosis [35].

**Table 2:** Lipid-related enzymes and bioactive lipids in exosomes

Lipid category	Lipid related enzymes	Functional effect(s)
LTA4, LTB4, LTC4	LTA4 hydrolase, LTC4 synthase	Triggering polymorphonuclear leukocyte migration
PGE2, 15d-PGJ2	COX-1, COX-2	Immunosuppression, PPARγ ligand
PGE2	PGE synthase	Inflammation
PA	PLD2, DGK	Increasing exosome production [
AA, LPC	cPLA2, iPLA2	Accounting for the membrane curvature
Phospholipase	sPLA2 IIA, sPLA2 V	Prostaglandin biosynthesis
Ceramides	nSMase2	Sorting cargo into MVBs
Cholesterol		Regulating exosome secretion
BMP		MVB formation and subsequent ILV biogenesis
PS		Being involved in exosome fate
SM		Triggering calcium influx
**References**		[36–41]

Traditional exosome isolation and purification methods mainly involve density gradient centrifugation [42, 43], size exclusion chromatography, and immunoaffinity purification [44, 45]. Ultracentrifugation is one of the most commonly used methods for exosome isolation [42, 46, 47]. This method is dependent on the fact that exosomes are smaller and denser than cells and can be separated by centrifugation at different speeds. Density gradient centrifugation is a separation method based on density differences of exosomes [42, 43]. In this method, fractionating macromolecules like sucrose, glucose, and proteins are poured into ultracentrifuge tubes. Exosomes localize and can be isolated -in a centrifuge tube according to different-sized macromolecules and their known density levels.

Size-exclusion chromatography is another separation method based on exosome size differences [48–50]. This method involves adding the sample to a size-exclusion chromatography column and using the filler to perform molecular screening so that larger cell fragments, proteins, and other molecules are screened while exosomes are discharged through the bottom.

Immunoaffinity purification is a separation method based on the binding affinity between exosome surface marker molecules (such as CD63, CD9, etc.) and specific antibodies [42, 51, 52]. This method mixes an affinity column containing specific antibodies with the sample and uses the binding force between the antibodies and labeled molecules to capture exosomes in a column, followed by elution and purification. This method has the advantages of solid selectivity and a good purification effect.

## Exosomes and cancer biology

### Exosomes promote tumor cell proliferation

Recent studies demonstrate cell-derived exosomes play an important role in tumor growth and progression [53]. For example, recombinant epidermal growth factor (EGF) treatment can promote the uptake of oral squamous cell carcinoma (OSCC) cell-derived exosomes into the OSCC cells. In contrast, EGF receptor (EGFR) knockdown or EGFR inhibitors, including erlotinib and cetuximab, abolishes exosome uptake by OSCC cells [53]. Cell-derived exosomes have also been shown to induce proliferation, migration, invasion, stemness, and chemoresistance of OSCC cells [53]. These biomolecules can activate signaling pathways and promote tumor cell proliferation by binding to receptors on the surface of tumor cells.

### Exosomes promote tumor cell metastasis

Extensive research on the tumor microenvironment has shown that cancer cell-derived exosomes are involved in crucial elements of cancer progression, including angiogenesis, premetastatic niche, extracellular matrix formation, epithelial mesenchyme transformation (EMT), cancer stem cell growth, and treatment resistance [54]. One study confirmed that cancer-associated fibroblast (CAF)-derived exosomes are a key driver in ovarian cancer (OVCA) tumor progression [55]. This study revealed specific circRNA (hsa_circIFNGR2) molecule can be used to elucidate the novel function of CAF-derived exosome circIFNGR2 on OVCA cell growth and metastasis. Metastatic organ tropism remains one of the greatest mysteries of cancer etiology since the "seed and soil" hypothesis. Scientists found that EGFR in exosomes secreted by gastric cancer cells can be delivered to the liver and integrated into the plasma membrane of liver stromal cells. Translocated EGFR has been shown to effectively activate hepatocyte growth factor (HGF) by inhibiting the expression of miR-26a/b. In addition, the upregulated paracrine HGF binds to the c-MET receptor on migrating cancer cells, providing fertile "soil" for "seeds" and promoting the landing and proliferation of metastatic cancer cells [36].

### Exosomes and the immune host response

During tumor growth, exosome components released by tumor cells can inhibit the normal function of the immune system, thereby avoiding attack and clearance by the host immune system. This inhibitory effect is mainly achieved by regulating the activity of immune cells, promoting immune cell apoptosis, and reducing the number of immune cells. The precise mechanism by which exosomes are involved in tumor progression remains elusive, multifaceted, and a double-edged sword, thus requiring further clarification. Exosomes can facilitate communication between innate immune cells and tumor cells to support or inhibit tumor progression. A past review focused on exosome-mediated intercellular communication between tumor cells and macrophages, neutrophils, mast cells, monocytes, dendritic cells, and natural killer cells [4].

### Exosome subtypes and tumor regulation

#### miRNA

microRNA can be used as a biomarker for the diagnosis of cancer. For example, pancreatic cancer is one of the leading causes of cancer death worldwide. Plasma microRNA is a promising player due to its non-invasive and practical use in tumor diagnosis and prognosis. Recent studies have shown that compared with healthy controls, microRNAs, namely miR-125b-3p, miR-122-5p and miR-205-5p, in plasma exosomes of pancreatic cancer patients are potential plasma exosome-derived of non-invasive biomarkers [56]. In addition to being used as a molecular marker for clinical diagnosis, microRNA in exosomes can also affect the growth of tumor cells by transferring into cells and changing their gene expression. For example, miR-142-3p is one of the miRs upregulated in various breast cancers. It activates the canonical Wnt signaling pathway and transactivates the expression of miR-150, leading to excessive proliferation of cancer cells in vitro and in vivo [57]. The miR in exosomes can also affect the tumor microenvironment. Communication between endothelial cells and tumor cells in the tumor microenvironment is required for cancer metastasis. Previous studies have confirmed that miR-29a expression is upregulated in colorectal cancer (CRC) tissues, and EMT-CRC cells may transport exosomal miR-29a to endothelial cells in the tumor microenvironment to increase vascular permeability, thus promoting the development and metastasis of CRC [58]. This process makes exosomal miR-29a a potential predictive marker for tumor metastasis and a viable therapeutic target for CRC. The miR in exosomes has an essential impact on tumor growth; therefore, future research requires further exploration on the mechanism of miR in exosomes on tumor growth and metastasis and develop new treatment strategies and markers.

### Proteins

Proteins (such as TTN-AS1, Rab27b) in exosomes can affect tumor growth through multiple pathways. First, they can directly act on cancer cells to promote proliferation and metastasis [59, 60]. Secondly, exosome proteins can affect the tumor microenvironment, indirectly affecting tumor growth [61]. For example, exosome proteins (SYT7) can promote tumor angiogenesis and provide an adequate supply of nutrients and oxygen, thereby promoting tumor growth and metastasis [62]. Third, some exosome proteins can regulate tumor immune response and inhibit the immune system's reaction to tumors, thereby promoting tumor evasion. Many proteins in exosomes related to tumors have been discovered [63], and these proteins have been confirmed as targets for cancer therapy.

### Lipids

Recent studies have shown that exosome lipids are important factors in tumor growth. These lipids mainly include phospholipids, cholesterol, and triacylglycerol. All these types of exosomes regulate tumor growth and metastasis through various mechanisms. First, exosome phospholipids can affect tumor cell growth by changing the composition and structure of cell membranes, thereby affecting signaling and membrane permeability. Enteropathogenic bacterial secretory particles were recently found to stimulate the intestinal epithelium to produce IDEN (intestinal mucosa-derived exosome-like nanoparticles), which contain elevated levels of sphingosine-1-phosphate, CCL20, and prostaglandins E2 (PGE2). As we know, gut-related inflammation plays a crucial role in the progression of colon cancer. In tumor immunity, it has also been found that the recruitment and proliferation of Th17 cells require CCL20 and PGE2, respectively. Demonstrated biological effects of sphingosine-1-phosphate contained in IDEN facilitate tumor growth in spontaneous and transplanted colon cancer mouse models [64]. In addition, phospholipids can serve as signaling molecules to participate in processes such as apoptosis and autophagy, thereby regulating tumor growth and metastasis. Triglycerides in exosomes can also affect tumor growth, since they are essential substances in lipid metabolism and can provide energy and nutrients for cell growth. Studies have shown that triglyceride synthesis and metabolic pathways in tumor cells are abnormally active, which may be related to their proliferation and metastasis capabilities.

## Emerging applications of exosomes in cancer diagnosis and therapy

### Exosomes as diagnostic tools

As previously mentioned, exosomes are present in many biofluids, which presents an opportunity to monitor cell status under normal or pathological conditions. In biopsies (liquid, plasma, serum, urine, saliva), exosomes have been used to analyze and determine a patient diagnosis, prognosis, progress, and chemoresistance status [65, 66]. Furthermore, up-regulated exosome secretion has been found in several complex conditions including cancer. Under pathological conditions, cellular changes can be monitored based on exosomes released by cells. Discrepancies in levels of specific molecules can be identified and analyzed by transcriptomics, proteomics, and lipidomic investigations [67]. The use of exosomes for disease diagnosis has been investigated in early diagnosis and prognosis of cancer [15–17] and cardiovascular diseases [68, 69].

Exosomes in blood samples for cancer detection has gained recent attention. In a retrospective study [16], researchers combined artificial intelligence and surface-enhanced Raman spectroscopy technology to successfully detect exosomes in early-stage cancers (lung, breast, and colon, liver, pancreatic, and gastric cancer). The study also includes a classification model to identify plasma exosome signaling patterns to identify their presence and tissue of origin. The final comprehensive decision model showed a sensitivity of 90.2% and a specificity of 94.4%, while predicting tumor organelles in 72% of positive patients. In colorectal cancer, exosomes have emerged as promising biomarkers for diagnosis due to their rich biological fingerprints and high stability. Recent research highlights the construction of an exosome enrichment platform on a 3D porous sponge microfluidic chip [15], whereby the exosome capture efficiency of the chip is approximately 90%. Deep mass spectrometry analysis and multilevel expression screening reveal a CRC-specific exosomal membrane protein (SORL1). A specific quantum dot-labeled SORL1 detection method was further designed. The systems demonstrated similar diagnostic performance in treating patients with early-stage CRC, young CRC, and CEA-negative CRC.

Compared with traditional biomarker detection methods, using exosomes for disease diagnosis has the following advantages: (1) High sensitivity: Exosomes can carry many biomarkers and, therefore, have high sensitivity. (2) High specificity: Exosomes can carry cell-specific biomarkers and therefore have high specificity. (3) Non-invasive: Exosome collection can be completed through conventional body fluid methods without invasive operations such as tissue sectioning or puncture. (4) Easy to store: The collected exosomes can be stored for a long time through cryopreservation and other methods to facilitate subsequent analysis. Although using exosomes for disease diagnosis has many advantages, several challenges still exist: (1) Standardization issues: Due to the current lack of unified exosome collection, processing, and detection standards, results may vary between different laboratories. (2) Technical issues: The current detection technology for exosomes is not mature enough, and new detection technologies need to be further developed. (3) Sample quantity issue: Since the current understanding of exosomes is not deep enough, many samples are needed for verification and confirmation.

### Exosomes in disease treatment

The application of exosomes in cancer treatment mainly depends on two strategies: (1): use as drug carriers to encapsulate drugs [70]; (2) use as bioactive molecules (e.g. miRs) to regulate the tumor microenvironment and inhibit tumor growth and metastasis. Demonstration studies have shown that using exosomes as drug carriers can improve drug inhibition and bioavailability and reduce drug side effects [71, 72]. This kind of RNA nanoparticles can improve the targeting ability and deliver therapeutic drugs to specific cancer cells. It has been reported that RNA nanoparticles can be used to display chemical ligands (such as folic acid [72, 73]), chemical drugs, or RNA aptamers on the surface of exosomes. These combinations can deliver small-molecule chemical drugs to a target via chemical ligands or RNA aptamers. By binding RNA nanoparticles to specific tumor cells, the dose and side effects of the drug can be reduced to achieve the purpose of targeted cancer treatment [74]. In addition, RNA nanoparticles modified on exosomes can also combine with siRNA[75, 76], miR [77–80], RNA aptamers, or ligands to specifically inhibit cancer cells.

## Exosomes in targeted drug delivery

Compared to liposomes and nanoparticle delivery systems which are synthesized in vitro, exosomes originate from the body and offer better biocompatibility and lower immunogenicity than their counterparts [81]. Furthermore, due to the heterogeneity of exosomes, they carry various proteins on the surface, which enter cells through a variety of ways after initial cell contact. Among them, receptor-mediated endocytosis is one main way information and drug transport can be achieved in target tissues [82].

Exosomes have been explored for their inherent ability to be loaded with both small and large molecules in support of their therapeutic use for treatment of various diseases [83]. The construction of engineered exosomes loaded with drugs can provide an efficient, less toxic, and more targeted drug delivery strategy. In addition, exosomes carry different peptides to target specific cells, which provides a strategy to construct more specific drug delivery mechanism. For example, Cui et al. engineered exosomes to deliver si*Shn3* for anti-osteoporosis functions in mouse models and demonstrated bone targeting capability in vitro and in vivo to achieve osteogenesis [84].

There are two main methods to encapsulate drugs in exosomes: natural and artificial. Natural packaging refers to using the biological properties of exosomes to package drugs inside, while artificial packaging refers to the application of chemical or physical methods to guide drugs into exosomes [85]. Both methods are described in detail below.

### Natural drug encapsulation

Exosomes have natural packaging properties and can encapsulate biomolecules inside and release them outside of cells. This natural wrapping mechanism is mainly achieved through modifying the cell membrane's protein, glycosylation, and lipid composition. Drugs can be encapsulated inside and released outside the cells by interacting with these biomolecules on the exosome membrane. The process of encapsulating drugs in exosomes is mainly achieved through the intracellular secretory pathway. Cells wrap drugs or other biomolecules in a membrane to form exosomes. These exosomes can be released by cells, enter the blood circulation system, and be transported to areas that require treatment [86]. The advantage of exosome-encapsulated drugs is that they can cross biological barriers, such as the blood–brain barrier, allowing drugs to reach hard-to-reach sites. In addition, exosomes can protect drugs from enzymatic degradation and immune system attack [87]. Exosomes can also achieve targeted delivery by modulating molecules on their surface. For example, in some diseases, molecules on the cell surface change, and these changes can be recognized by exosomes. Therefore, scientists can change the specific molecules, including drugs, on the surface of exosomes so they can be delivered to specific cells or tissues [88, 89]. In addition, exosomes can also achieve different delivery effects by changing their size and shape [24]. For example, smaller exosomes can pass through biological barriers more efficiently, while spherical exosomes can be more easily engulfed by cells. However, there are still some challenges and problems with exosome-encapsulated drugs. Controlling the speed and location of drug release from exosomes remains a complex issue, and the yield and purity of exosomes also need further improvement [25].

### Artificial drug encapsulation

Artificial encapsulation refers to guiding drugs into exosomes through chemical or physical means. This method can effectively improve drug packaging efficiency and control drug release speed. At present, there are mainly the following methods for manual packaging. (1) Electroporation method: Electroporation refers to mixing exosomes and drugs and using an electric field to cause the drugs to penetrate the exosome membrane and enter the interior. This method can achieve efficient drug encapsulation and release but requires high technical requirements and equipment support [26]; (2) Chemical cross-linking method: The chemical cross-linking method refers to using cross-linking agents to cross-link drugs with exosome membranes to achieve drug encapsulation and release. This method can control the drug release speed and time and realize the simultaneous encapsulation of multiple drugs [37]; Thermal shock method: The thermal shock method refers to mixing exosomes with drugs and then changing them through high or low-temperature treatment to achieve the encapsulation and release of drugs. This method is simple to operate but requires precise temperature and time control [38].

Recent studies have examined the therapeutic viability of loaded exosomes and whether cargo targeted delivery to the intended site of action works [90]. One group utilized drug-resistant A2780/DDP cells to test effectiveness of cisplatin-loaded exosomes against cisplatin alone to compare and determine therapeutic viability [91]. The study found that by using cisplatin-loaded exosomes, the cytotoxicity of cisplatin increased by factor of three in drug resistant cells and a factor of 1.4 in drug sensitive cells versus cisplatin alone. Another study tested therapeutic viability through loading blood-derived exosomes with dopamine for treatment of Parkinson’s Disease [92]. The authors found there was greater than 15-fold increase in bioavailability of dopamine in the brain through use of exosome delivery platform. As previous research has shown, the delivery of loaded exosomes can prove to be efficient in drug therapy that potentially avoids challenges that other platforms have encountered including first pass metabolism, limited bioavailability, and blood brain barriers.

## Strategies in exosome cargo loading

**Fig. 2:**
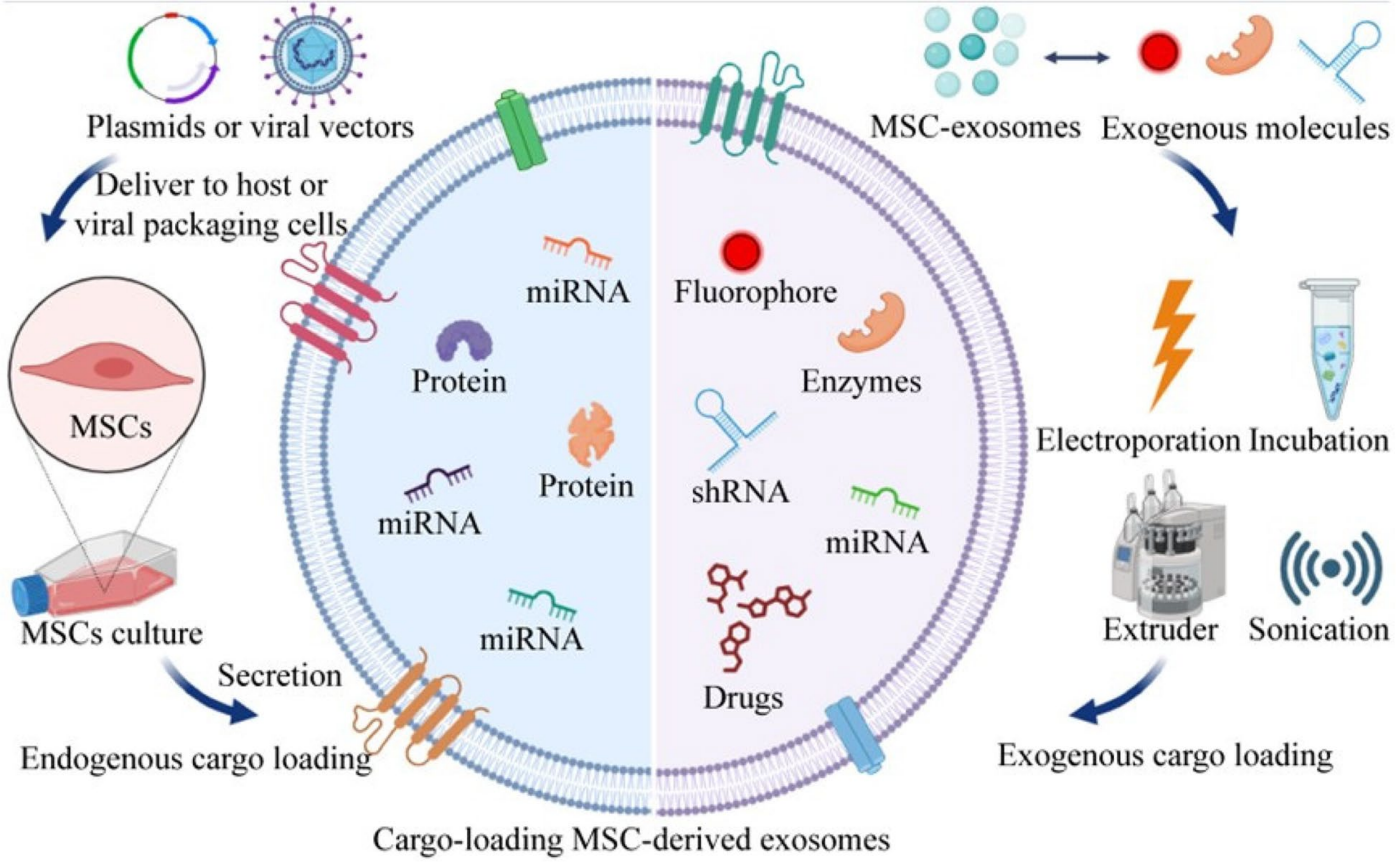
Cargo loading methods for exosome therapeutics. Endogenous cargo delivery with plasmids or viruses involves constructing transgenes and expressing them in vector cells, and then collecting endogenous cargo-packaged exosomes from the cell culture supernatant. These exosomes can express special antigenicity. Endogenous cargo loading often involves miRNAs and proteins with therapeutic efficacy. In exogenous cargo loading, delivery of drugs or therapeutic small molecules involves electroporation, co-incubation, or ultrasound to carry exogenous cargo

Due to their high biocompatibility, stability, and low immunogenicity, exosomes have been considered favorable carriers for drug delivery. The lipid bilayer membrane of exosomes also offers protection against cargo degradation, making them a desirable candidate for drug delivery. Several cargo delivery strategies exist involving small-molecule, nucleic acid, and protein drugs (Fig. 2). Recent studies have focused on modifying various aspects of exosome to substantially enhance their targeting ability.

Phospholipid modification: Since exosomes mainly comprise phospholipids, phospholipid modification has become a common strategy. Changing the type and content of phospholipids on the surface of exosomes can affect their stability, targeting, and immunogenicity [39]. For example, the blood–brain barrier (BBB) precludes the entrance of most pharmaceutical drug compounds into the brain. Clinically, delivery across BBB tight junctions is achieved through various endogenous transport mechanisms. Therefore, receptor-mediated transcytosis (RMT) through exosomal cell membrane components and BBB cells is one of the most widely studied and used methods to allow delivery of drugs into the brain. A variety of strategies exist involving use of exosomes to can express specific proteins through plasmids or via mechanical coating on exosomes Receptors that mediate transcytosis bind specific ligands to hijack RMT, such as transferrin receptor (TfR), low-density lipoprotein receptor (LDLR), and insulin receptor (INSR). Cell-penetrating peptides and viral components derived from neurotropic viruses can also be used for effective BBB crossing of therapeutic drugs [40].

**Fig. 3:**
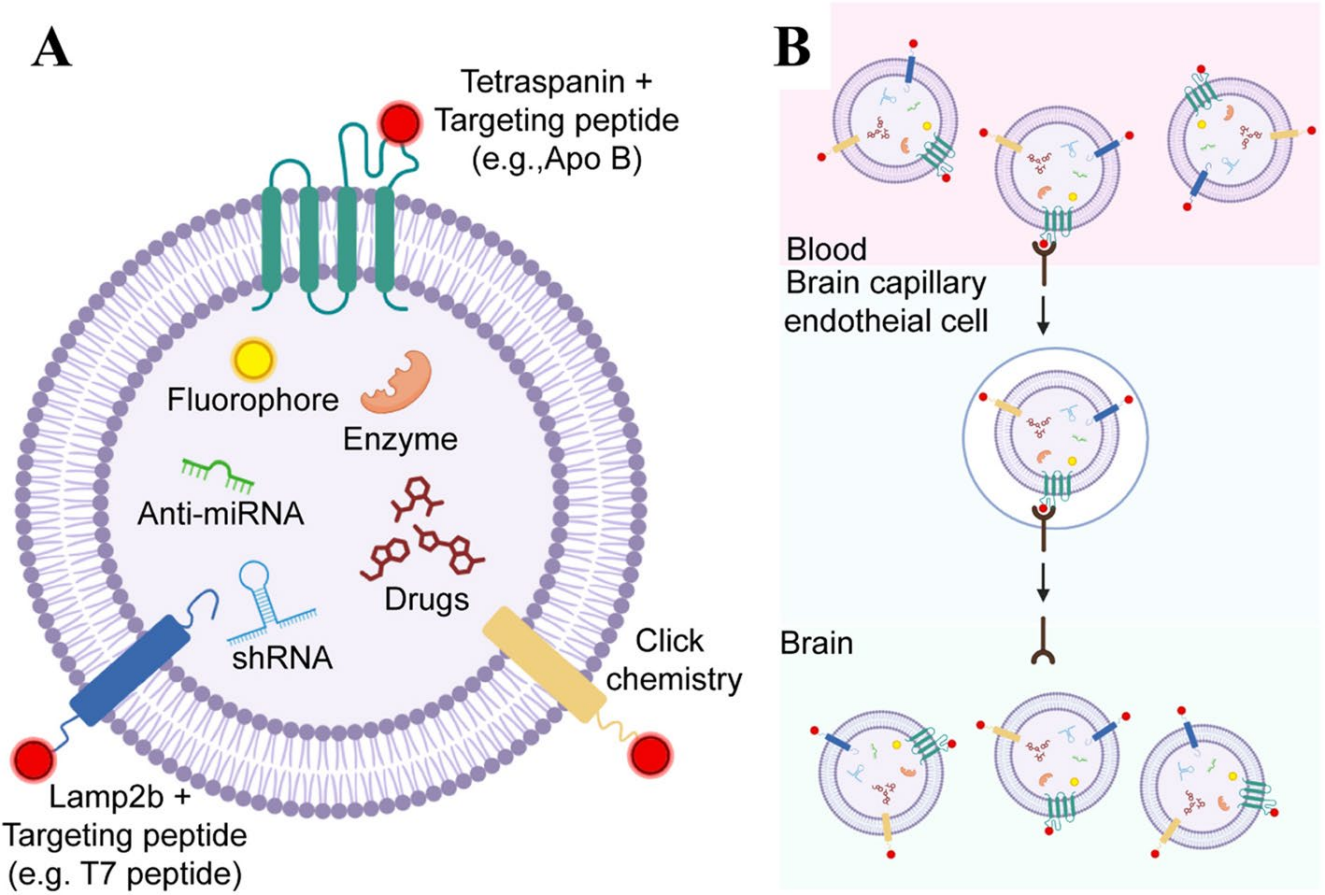
Chemical and protein modifications on exosomes to improve targeting efficacy. **A** Synthetic chemicals (such as folic acid) are combined with exosomes through chemical modification (click chemistry). The drug-carrying exosomes can be targeted to the receptors of cancer cells (such as folate receptors) through folic acid. Targeting peptides can also be fused to express (such as Lamp2b and tetraspanins) through genetic modification of exosome-producing cells (as described in Fig. 2). These synthetic short peptides can cross the blood–brain barrier. **B** Receptor mediated transcytosis (RMT) is a widely used method to allow exosomes to pass through blood brain barrer tight junctions. Surface-modified exosomes possessing brain targeting have shown enhanced CNS delivery

Protein modification: Proteins on the surface of exosomes can be modified through glycosylation, acetylation, phosphorylation, etc. These modifications can affect exosome targeting, stability, and immunogenicity properties. Previous study have demonstrated modifying exosome surface proteins can affect their interaction with the liver microenvironment, thereby promoting gastric cancer metastasis to the liver [36]. In other studies, targeted delivery of exosomes to the brain can be achieved by labeling various targeting moieties on the surface of exosomes. Therapeutic exosomes can be engineered through chemical modifications (e.g., click chemistry) to express various targeting moieties or genetic modification of exosome-producing cells to express targets fused to exosome membrane-associated components toward peptides such as Lamp2b and tetraspanins (Fig. 3) [41].

Chemical decoration: The properties of the exosome surface bilayer can be modified by cholesterol-binding RNA nanoparticles. These RNA nanoparticles can be used to display chemical ligands (such as folic acid), chemical drugs, or RNA adapters on the exosome surface. body. Folate receptors are abundantly expressed on many human tumor cells. Therefore, folic acid modified on the surface of exosomes can improve the targeting ability of RNA nanomedicines and deliver therapeutic drugs in exosomes to specific cancer cells [72, 73].

## Case study: exosomes and siRNA nanotechnology

Ribonucleotide reductase M2 subunit (RRM2) is a dimer containing a tyrosine free radical and a non-heme iron, essential for enzyme activity for DNA synthesis and damage repair [93, 94]. When DNA replication occurs, RRM2 is expressed during late G1/early S phase. Overexpression of RRM2 enhances the metastatic and invasive capacity of human cancer cells [95–97] and leads to poor prognosis in patients [98, 99]. RRM2 also promotes tumor angiogenesis through regulating expression of proangiogenic factor VEGF and antiangiogenic factor TSP-1 [100]. Previous reports indicated that the antisense molecules to RRM2 effectively decreases RRM2 expression, inhibits enzyme activity and reduces growth of cancer cells in vitro and in vivo [101–103]. Here, to leverage the targeting effect and therapeutic potential of folate RNA aptamer for tumor treatment, we designed an arrow-tail RNA nanoparticle to display folate RNA aptamer on the surface of exosomes to deliver M2 siRNA folate RNA Apt/Exo/siM2 for cancer treatment.

In the following case study, we employed RNA nanotechnology to display RNA ligand on exosomes to serve as a carrier for siRNA. An RNA ligand that was anchored on exosomes by cholesterol targets folate receptors to recognize cancer cells [104, 105]. Once entry into the cancer cells was achieved, siRNA silenced the anti-apoptotic factor M2 subunit of ribonucleotide reductase (RRM2). In comparison with other delivery systems with the cyclodextrin-containing polycation nanoparticles to protects siRNA [106], a human transferrin protein (hTf) ligand was displayed on the nanoparticle to target Tf receptors (hTfR) on the cancer cells [107]. The siRNA carried by the nanoparticle also silences RRM2 [108]. Based on in vivo results, the exosome-delivered siRNA silencing RRM2 demonstrated noticeable melanoma growth regression, suggesting the RNA-targeted exosome delivery system with RRM2siRNA could attenuate tumor growth.

### Suppression of melanoma and colorectal carcinoma growth in vivo by FOLATE_Apt_/Exo/siM2

**Fig. 4:**
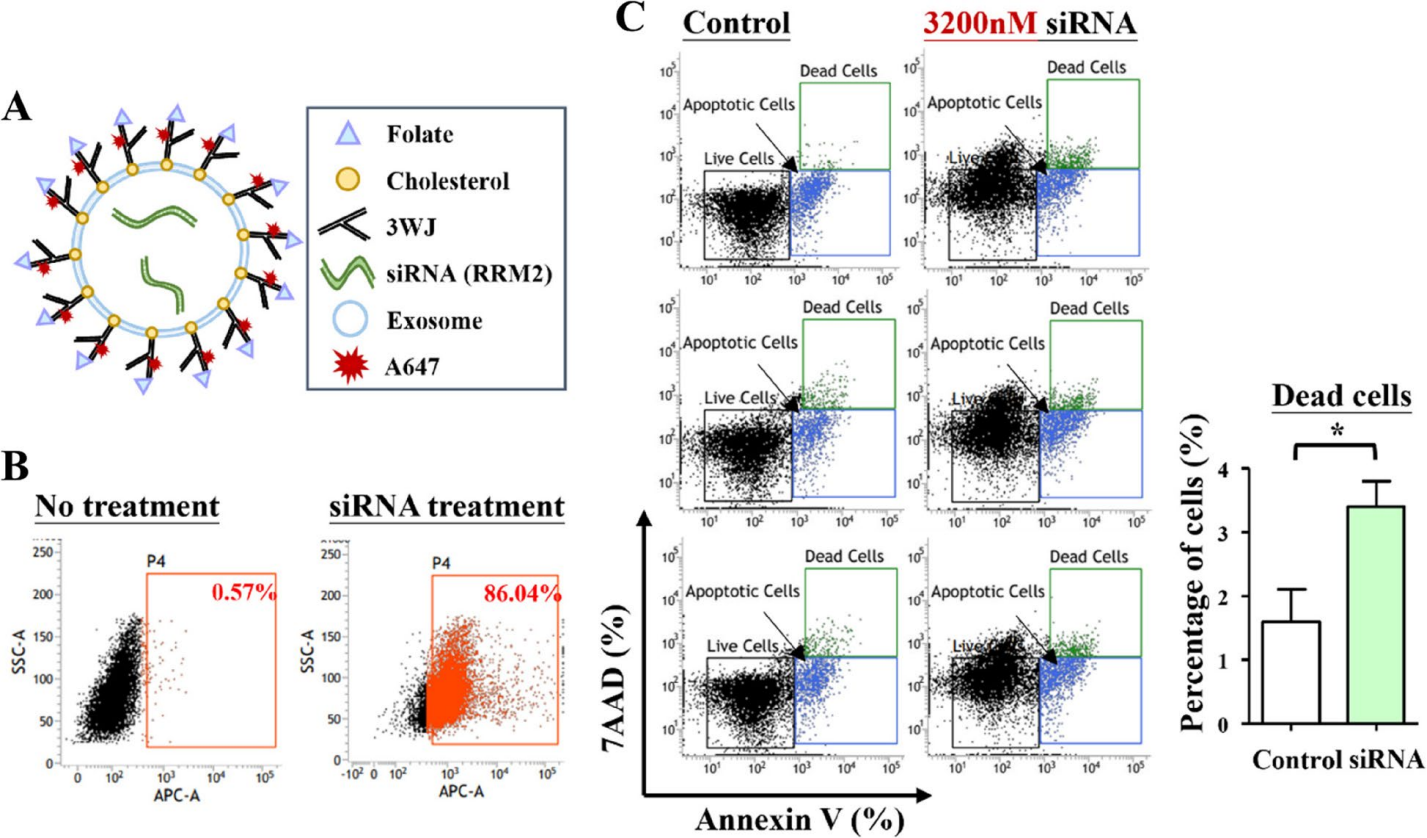
Exo/siM2 fused with cells when A375 cells were incubated with Alexa647 labeled-RNA for 1 h. **A** The construction of exosomes that display the RNA/folate complex with Alexa647. **B** Flow cytometry result. After A375 cells incubated with 50 nM Alexa647 labeled-RNA loaded exosome, high percentage of fluorescence positive cells were detected. **C** A375 cells were incubated with 3200nM Exo/siM2 and **D** exhibited a significant increase of dead cells and apoptotic cells. The results are presented as the mean ± SD. *P < 0.05

We chose melanoma cell lines, A375, for investigation because of the high RRM2 mRNA expression level. A375 cells were incubated with 50 nM Alexa647 labeled-RNA loaded exosome (Fig. 4A). Approximately 86.04% ~ 92.47% of fluorescence positive cells were observed (Fig. 4B). Flow cytometry of A375 cells treated with 3200nM of FOLATEApt/Exo/siM2 demonstrated the increase of the dead cell population in the treated cell line in contrast to the control (Fig. 4C) while the apoptotic cell populations did not differ between the treated and the control (Fig. 4C). Thus, FOLATEApt/Exo/siM2 causes cancer cell death in vitro.

**Fig. 5:**
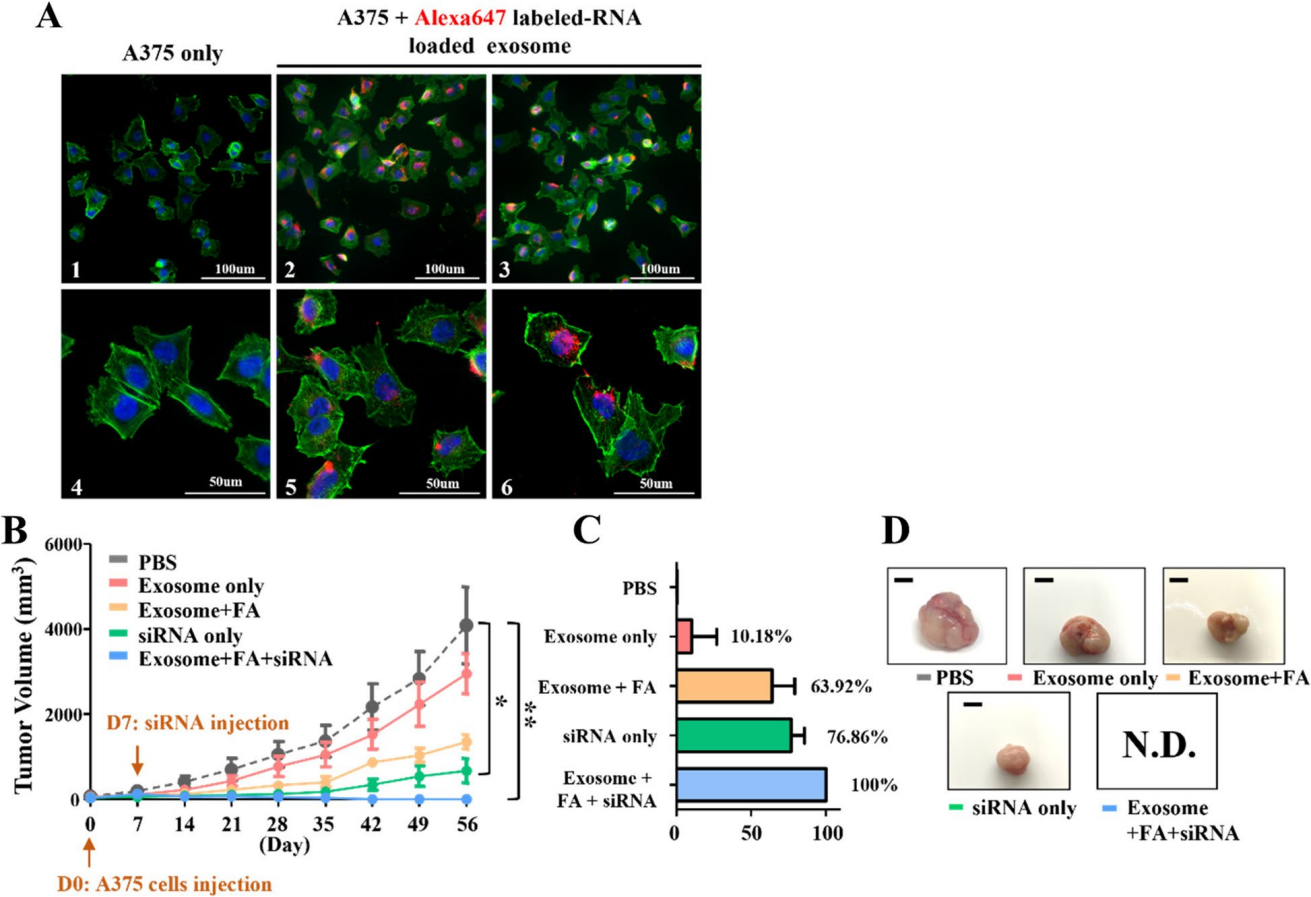
Tumor growth and inhibition rate (TGI) of A375 tumor in NSG mice. **A** Cell fluorescence image. (1) and (4), the control group, were the A375 cells staining with Alexa Fluor® 488 phalloidin for cytoskeleton (green) and DAPI for nucleus (blue). (2)(3)(5)(6) were the A375 cells cocultured with Alexa647 labeled-RNA loaded exosome (red) for 1 h and stained with Alexa Fluor® 488 phalloidin for cytoskeleton (green) and DAPI for nucleus (blue). **B** Tumor growth curve of different treated groups. **C** Tumor growth inhibition rate (TGI) of different treated groups. **D** Grow images of A375 tumors after treatment for 49 days. The results are presented as the mean ± SD. *P < 0.05, **P < 0.01

Fluorescent microscopy of A375 cells showed the red fluorescence inside the cells, indicating FOLATEApt/Exo/siM2 entered the cells (Fig. 5A). The in vivo melanoma targeting and anti-tumor efficacy of FOLATE_Apt_/Exo/siM2 were investigated in a tumor xenograft NSG mouse model. The results indicates that the fully packaged exosomes with folate display and siRNA load reduced tumor volume by 100%, compared to siRNA by 77% and folate by 64%. The inhibition was FOLATE_Apt_/Exo/siM2 packaging-dependent (Fig. 5B-D). Therefore, the packaging-dependent tumor suppression by FOLATE_Apt_/Exo/siM2 demonstrates that complete packaging of exosomes with folate, RNA aptamer, and siRNA reduced cancer cell growth.

**Fig. 6:**
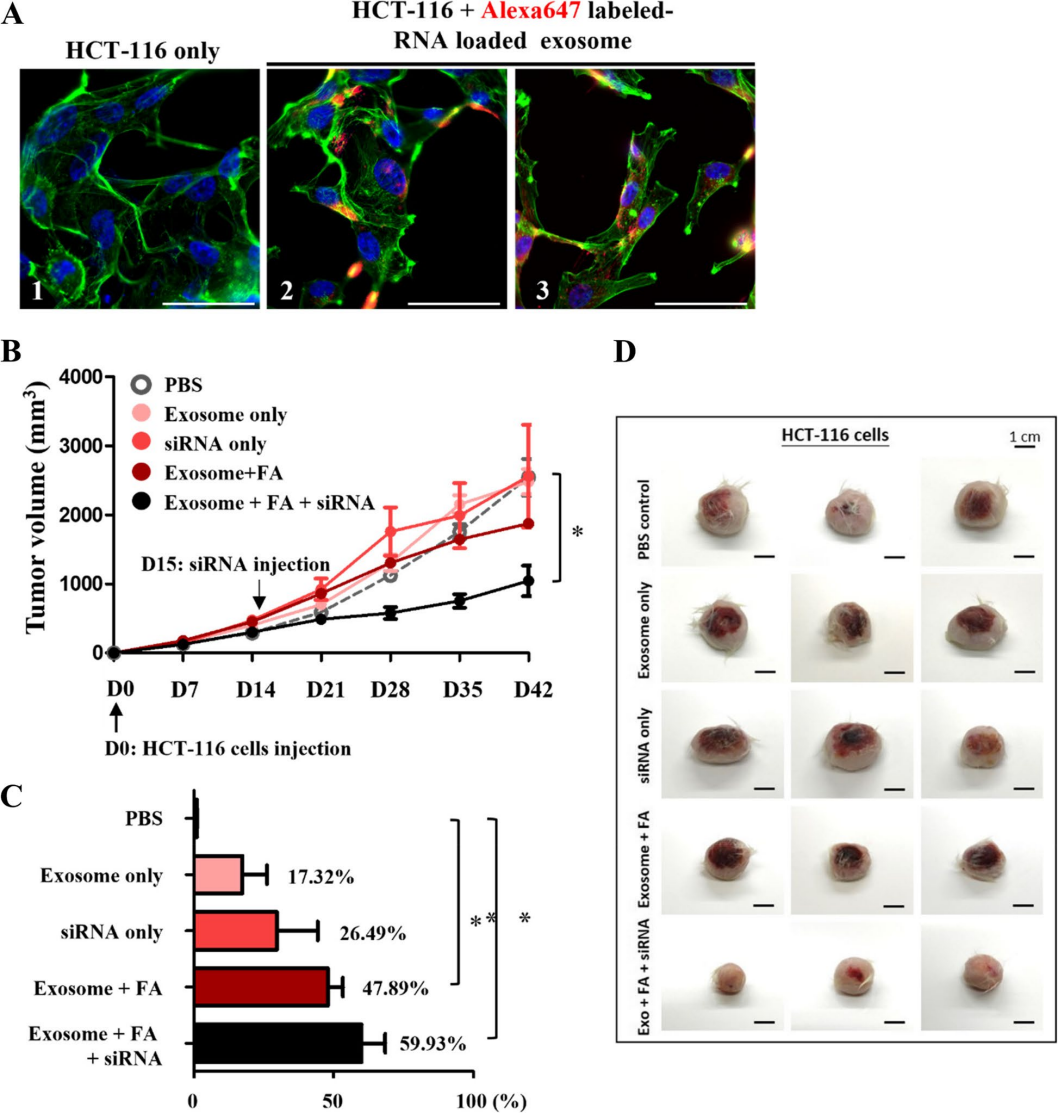
The growth and suppression rate of HCT-116 tumor in NSG mice. **A** (1)(2)(3) were colorectal tumor (HCT-116). (1)(4), the control groups, were cells staining with Alexa Fluor® 488 phalloidin for cytoskeleton (green) and DAPI for nucleus (blue). (2)(3) were the cells cocultured with Alexa647 labeled-RNA loaded exosome (red) for 1 h and stained with Alexa Fluor® 488 phalloidin for cytoskeleton (green) and DAPI for nucleus (blue). **B** Tumor growth curve of different treated groups. **C** Tumor growth inhibition rate (TGI) of different treated groups. **D** The end of experiment, the HCT-116 tumors were harvested for gross-view observation. The results are presented as the mean ± SD. *P < 0.05

With FOLATE_Apt_/Exo/siM2 packaging-dependent suppression of melanoma established, FOLATE_Apt_/Exo/siM2 suppressed other cancer cell lines as well. Fluoresce microscopic images of malignant colorectal carcinoma cells incubated with FOLATEApt/Exo/siM2 suggest that the exosomes appeared localized in nuclei (Fig. 6A) where transcription occurs. Growth curve of the tumor derived from the colorectal carcinoma cell line HCT-116 treated with FOLATEApt/Exo/siM2 and the packaging controls exhibited FOLATE_Apt_/Exo/siM2 packaging-dependent inhibition (Fig. 6B-D). The results show that the wholly packaged exosomes with folate display and siRNA load decreased tumor volume by 60%, compared with siRNA by 27% and folate by 48% (Fig. 6C).

## Clinical applications and current clinical trials involving exosomes

### Current clinical trials in exosomes

In recent years, exosomes have been investigated in clinical trials in a variety of contexts and applications. A survey of the current clinical trial landscape from GlobalData database (www.globaldata.com) reveals the biggest areas that exosomes are being explored for clinical therapies are oncology (54%), central nervous system (13%), infectious disease (13%), and immunology (8%) (Table 3). Of the 420 ongoing exosomal drugs in clinical development, more than 65% of the products remain in early phase (Preclinical to Discovery) and not yet filed for independent new drug review by U.S. Food and Drug Administration, while only 6.4%, 7.3%, and 2.3% of them are in Phase 1, 2, and 3 clinical trial, respectively. To date only two products have been marketed for clinical use. First, Patisiran is indicated for treating polyneuropathy associated with hereditary transthyretin-mediated amyloidosis (hATTR) in adults. Patisiran is a first-of-its kind RNA interference (RNAi)-based therapy designed to reduce levels of both WT and mutant transthyretin (TTR) for the treatment of patients with hereditary transthyretin-mediated amyloidosis, a congenital neurological and cardiac disorder [109]. Ibudilast is another commercially available exosome-related product. It is a relatively nonselective phosphodiesterase inhibitor which has been marketed for almost 20 years in Japan for treating asthma [110]. More recently it has been found to have anti-inflammatory activity in both the peripheral immune system and in the CNS via glial cell attenuation in relapsing–remitting (RR) and/or secondary progressive (SP) multiple sclerosis [111].

**Table 3:**
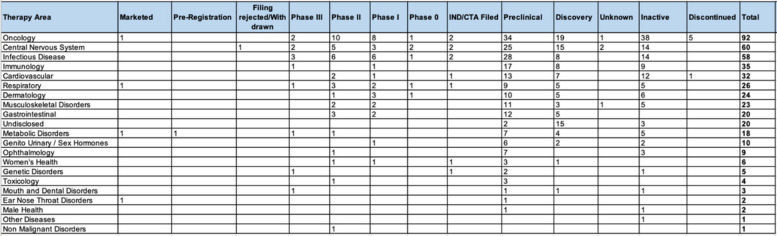
Number of exosome drugs according to indication/therapeutic area

The application of exosomes in the context of treating neurological diseases and cardiovascular disease has attracted recent attention. While there is currently no effective treatment for neurological diseases such as Alzheimer's disease and Parkinson's disease [112], certain bioactive molecules in exosomes can promote the growth and repair of neurons and are expected to become a new way to treat neurological diseases. For example, researchers have found that neurotrophic factors contained in exosomes can promote the growth and repair of neurons [113]. They injected exosomes into the brains of mice and found that exosomes could promote the growth and repair of neurons and improve the cognitive function of the mice. In cardiovacular research, pathologies like such as coronary heart disease and myocardial infarction are some of the leading causes of death worldwide. Bioactive molecules (such as microRNA-132), which are delivered by mesenchymal stem cell-derived exosomes, promote angiogenesis in myocardial infarction [114]. Researchers have found that miR-126 contained in exosomes can promote endothelial cell proliferation and angiogenesis and reduce vascular permeability. They encapsulated miR-126 in exosomes and injected it into the myocardial infarction site of mice. They found that exosomes could promote the proliferation and repair of cardiomyocytes too improve the heart function of mice [115].

Regardless of their clinical indication, exosomes and their usage need to comply with good manufacturing practice (GMP). A GMP grade exosome production process comprises the highest quality of materials, cells, culture environment, manufacturing technologies, and skilled workforce for highly controlled and strictly monitored conditions. Furthermore, purification and quality control release assays are essentials after production to ensure final product meet highest standards prior to administration to patients for intended use. The European Medicines Agency and US Food and Drug Administration have convened workshops and acknowledge new scientific progress in cellular and molecular biotechnology have led to the development of advanced therapies. Both agencies have also released recommendations and classifications for advanced therapy production. As the nascent field of exosomes continue to expand in the realms of therapy and diagnostics, further recommendations, guidelines and criteria for researchers and manufactures are warranted to keep advanced therapies safe for patient use.

## Future development of exosomes in therapeutics

### Precision modification technology

With the development of precision gene editing technology, precise modification of exosomes can be achieved through genetic engineering in the future, thereby achieving more precise regulation [112]. Previous study introduces the use of precision gene editing technology to precisely modify exosomes to achieve therapeutic effects on Parkinson's disease. Genetic engineering allows therapeutic vectors to be loaded into exosomes and treated by implanting them into cells. The article discusses the application prospects and challenges of exosomes in the field of neurology.

### Cell engineering technology

Cell engineering technology can control the cells from which exosomes are derived, thereby affecting their secretion quantity and quality [116]. Another study introduces cell engineering technology to control the cells from which exosomes are derived, thereby affecting exosome secretion quantity and quality. Scientist explores the application of human umbilical cord mesenchymal stem cell exosomes to promote angiogenesis through the Wnt4/β-catenin pathway and introduces the application prospects of cell engineering technology in controlling exosome secretion.

### Nanotechnology applications

Nanotechnology offers enormous potential to further explore and impact cell physiology and pathology [117]. Recent studies describes the use of nanotechnology to control the size, shape, and surface properties of exosomes to influence their effects on physiology and pathology. The article explores the potential of using nanotechnology to change the properties of exosomes and apply them to cancer treatment [117].

In summary, exosomes are extracellular vesicles with great potential and play essential roles in physiology and pathology. By modifying it in different ways, its biological activity and stability can be controlled, enabling in-depth research and application of its function and mechanism of action. With the continuous development of technology, exosome modification technology will be more widely used.

## Conclusion

Due to their clinical potential and unique biological functions, exosome offer potential breakthroughs in drug delivery, noninvasive disease diagnosis, treatment, and other fields. Compared to liposomes, nanoparticles, microspheres, microemulsions, and other synthetic drug loading systems, exosomes pose natural and unique advantages as potential biomarkers for prognosis and diagnosing disease, drug delivery carriers, cell free therapy and cancer vaccine.

Considering the promise of exosomes in diagnosing and treating various conditions, challenges remain for its translation from the lab to the clinic. First, there are limitations surrounding vesicle isolation and determining whether the isolated exosomes are viable candidate for clinical use. This can be due to multiple factors including inconsistencies related to the isolated particle number, morphology, methodology, conditions, and source of isolated exosomes. Another prominent limitation is variability in exosomal yield and purity related to isolation techniques. While some isolation techniques can provide higher yield and purity than others, inconsistencies in methodology, equipment, and human error prove it is difficult to quantify which method is most appropriate to use. Alongside this are inconsistencies in cargo loading efficiency such as electroporation, sonication, and extrusion.

While exosome research remains in its nascent stages of development, further in-depth understanding of subcellular components and mechanisms involved in exosome formation and specific cell targeting will bring light on their physiological function and clinical potential. Undoubtedly, exosomes represent a promising tool in the field of medicine and may provide a solution to a variety of medical challenges we face today.

## Data Availability

Not applicable.
